# Generation and Characterization of an *Nse*-*CreER^T2^* Transgenic Line Suitable for Inducible Gene Manipulation in Cerebellar Granule Cells

**DOI:** 10.1371/journal.pone.0100384

**Published:** 2014-06-20

**Authors:** Theresa Pohlkamp, Laura Steller, Petra May, Thomas Günther, Roland Schüle, Michael Frotscher, Joachim Herz, Hans H. Bock

**Affiliations:** 1 Center for Neuroscience, Department of Neuroanatomy, Albert-Ludwigs-University, Freiburg, Germany; 2 Department of Molecular Genetics, University of Texas Southwestern Medical Center, Dallas, Texas, United States of America; 3 Clinic for Gastroenterology, Hepatology and Infectiology, Heinrich-Heine-University, Düsseldorf, Germany; 4 Department of Urology, University Hospital Freiburg, Freiburg, Germany; 5 Institute for Structural Neurobiology, Center for Molecular Neurobiology, Hamburg, Germany; Seattle Children's Research Institute, United States of America

## Abstract

We created an *Nse*-*CreER^T2^* mouse line expressing the tamoxifen-inducible CreER^T2^ recombinase under the control of the neuron-specific enolase (*Nse*) promoter. By using Cre reporter lines we could show that this *Nse*-*CreER^T2^* line has recombination activity in the granule cells of all cerebellar lobules as well as in postmitotic granule cell precursors in the external granular layer of the developing cerebellum. A few hippocampal dentate gyrus granule cells showed Cre-mediated recombination as well. Cre activity could be induced in both the developing and adult mouse brain. The established mouse line constitutes a valuable tool to study the function of genes expressed by cerebellar granule cells in the developing and adult brain. In combination with reporter lines it is a useful model to analyze the development and maintenance of the cerebellar architecture including granule cell distribution, migration, and the extension of granule cell fibers *in vivo*.

## Introduction

The cerebellum plays an important role in motor control and is involved in cognitive processing and motor learning [1], [2]. The small-sized granule cells (GC) in the cerebellum are the most abundant neurons in the mammalian brain. They are densely packed into a thick layer of the cerebellar cortex, the GC layer (GCL). Next to the GCL of the trilaminated cerebellum, the cell bodies of the Purkinje cells form the Purkinje cell layer. The most superficial layer is the molecular layer (ML), which mainly consists of dendritic and axonal fibers. Amongst these fibers are Purkinje cell dendrites, innervated by climbing fibers (projections from the inferior olivary nucleus of the medulla oblongata) and parallel fibers (the axons of the vast number of the GCs). Moreover, the ML harbors two types of small inhibitory interneurons (basket and stellate cells). During development most cerebellar neurons originate in the ventricular zone of rhombomere 1, except GCs [3]. In the mouse, the vast majority of proliferating GC-precursors (GCPs) start to arise at the rhombic lip between embryonic day 13 (E13) and E16. Some deep cerebellar neurons arise between E9 and E11, even before GCs are generated. From there GCPs migrate tangentially to form the external granular layer (EGL), a secondary proliferative zone beneath the cerebellar pial surface [4]–[7]. GCP proliferation in the outer EGL continues until approximately postnatal day 15 [8]. The GCPs exit their cell cycle and start differentiating by migrating into the inner part of the EGL. Postmitotic GCPs in the deep EGL extend bipolar processes, which will become parallel fiber axons, and migrate tangentially – in parallel to the pial surface – before they turn orthogonally, to migrate radially along Bergmann glial fibers. After passing the ML and the Purkinje cell layer, GCs reach the deeper internal granular layer (IGL), which later becomes the mature GCL [3], [9].

CreER^T2^ is a fusion protein of the bacteriophage P1 recombinase (Cre) and the estrogen receptor (ER) whose ligand binding-domain was modified to bind tamoxifen (ER^T2^ [10]). Tamoxifen forces the dissociation of the ER^T2^-bound heat shock protein 90 (HSP90), allowing CreER^T2^ to translocate into the nucleus. In the nucleus Cre(ER^T2^) binds to LoxP-sequences and deletes LoxP-flanked genomic parts via recombination. Hence, the Cre(ER^T2^)/LoxP-system is a useful tool to modify gene expression in a temporally and spatially controlled manner.

To enable neuron-specific *CreER^T2^* expression, the neuron-specific enolase (*Nse*) promoter was chosen to drive *CreER^T2^* transcription. Microinjection of the cloned *Nse-CreER^T2^* cassette yielded one transgenic mouse line (*Nse*-*CreER^T2^*). To characterize Cre recombinase activity after tamoxifen injection, two different reporter lines were used, expressing β-Galactosidase (β-Gal, also known as LacZ) or membrane-tagged GFP (mGFP) after recombination. The results show that CreER^T2^-recombinase in this mouse line enables a tamoxifen-inducible recombination of LoxP-flanked genomic regions that is specific to the GC population in the cerebellum. In addition, a few hippocampal dentate gyrus granule cells also showed inducible Cre activity. Some fibers crossing the colliculus superior in the midbrain and the medial vestibular nuclei in the medulla were mGFP-postive as well.

To our knowledge, this is the first described mouse line with abundant *CreER^T2^* expression in the GCL of all cerebellar lobules, restricted to the GC population. Targeting gene manipulation to cerebellar GCs in a temporally specific manner provides a powerful tool for investigating the function of proteins expressed or secreted by GCs, as well as a means of fate-mapping cerebellar GCs. This will lead to novel insights into the molecular and cellular events of postnatal development and maturation of the cerebellum, as well as GC function in the adult brain.

## Material and Methods

### Ethics Statement

All experimental procedures were performed according to the approved institutional guidelines for animal care at the University of Freiburg (license no. 35-9185.81; G-08/33) or University of Texas Southwestern Medical Center (IACUC license no. 0701-07-01-4).

### Generation of an *Nse-CreER^T2^* Transgenic Mouse Line

**Figure 1:**
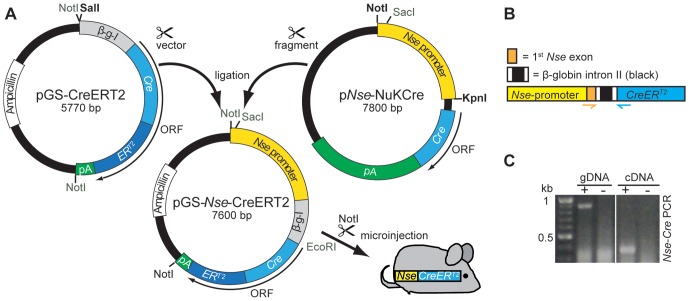
Cloning of pGS-*Nse*-*CreER^T2^* and transgenic cDNA detection in *Nse-CreER^T2^*. A: After linearizing (SalI, blunted) the pGS-*CreER^T2^* vector, it was ligated with the *Nse*-promoter fragment of the p*Nse*-*NukCre* plasmid (cut with NotI and KpnI, blunted). To prove the correct orientation of the fragment, a PCR was performed. The *Nse*-*CreER^T2^* transgenic cassette (cut with NotI) was used for pronucleus injection. B: Schematic illustration of the transgenic cassette. The chicken β-globin intron II (black, flanked by fragments of the β-globin exons II and III, white) is located between the *Nse*-promoter (yellow, part of first non-coding *Nse*-Exon in orange) and *CreER^T2^*. To detect the spliced transcript an *Nse-Cre*-PCR was designed where the forward primer is located in the *Nse*-exon and the reverse primer in the *Cre* encoding region (orange and blue arrows, respectively). C: Amplification (+) of genomic DNA (gDNA, 900 bp) and transcript cDNA (350 bp), wt genomic/cDNA was used as negative control (−).

To generate pGS-*Nse*-*CreER^T2^*, the *Nse*-promoter fragment of p*Nse-NuKCre* [11] was excised using KpnI and NotI. After filling in the restriction sites using Klenow fragment (NEB, DNA Polymerase I, Large Klenow Fragment) the promoter was cloned into the SalI-opened, Klenow-treated and dephosphorylated (Alkaline Phosphatase, NEB) pGS-*CreER^T2^* acceptor-vector [12] (**Fig. 1A**). The orientation of the promoter was verified by SacI and EcoRI restriction digest and PCR (see below).

For microinjection pGS-*Nse*-*CreER^T2^* was cut with NotI and the *Nse*-*CreER^T2^* cassette was isolated via gel electrophoresis and gel-extraction (Qiagen). The DNA was eluted in 5 mM Tris, pH 7.5 with 0.1 mM EDTA and microinjected into pronuclei of fertilized FVB oocytes using standard procedures. The resulting founder transgenic mouse was transferred to a specific pathogen free (SPF) housing. Breeding of wild type (C57/BL6 Background) and transgenic animals was conducted in accordance with institutional guidelines. Tail biopsies were digested with proteinase K (Applichem) and genotyping was performed by *Nse*-*Cre* PCR (see next section) to screen for transgenic founder animals and for routine genotyping. The line has been deposited at Jackson Laboratories (Jax Stock 022763).

### *Nse-Cre* PCR, Genotyping

PCR was performed using transgene-specific primers that bind within the *Nse*-promoter and the Cre-coding regions (forward *Nse*-primer sequence 5′-CGT CAC CAC CGC CAC CGC CAC-3′; reverse *Cre*-primer sequence 5′- ACG ACC GGC AAA CGG ACA GAA GCA-3′). The reaction mixture contained 67 mM Tris (pH 8.8); 16.6 mM (NH_4_)_2_SO_4_; 6.7 mM MgCl_2_; 6.7 µM EDTA; 0.0348% β-mercaptoethanol; 10% DMSO; 0.1 mg/ml BSA; 1.25 mM dNTPs (each); 1% Taq polymerase; 500 nM primer (each); and template (final reaction volume 20 µl). The PCR started with 2 min at 94°C, 2 min at 55°C and 3 min at 67°C, followed by 40 cycles (94°C for 30 s, 55°C for 30 s, and 67°C for 60 s) and a final elongation step of 10 min at 67°C. PCR products (916 bp) were visualized by separation on an 1% agarose gel and staining with ethidium bromide.

### Detection of *CreER^T2^* Transcripts via RT-PCR

Whole brain RNA was extracted using the TRIzol method (Invitrogen). After DNase treatment (Fermentas) 2 µg of RNA were reverse transcribed (M-MLV-RT, Promega) into cDNA, using random primer mix (Promega). To detect the *CreER^T2^*-transcript an *Nse*-*Cre*-PCR was performed. The *Nse*-*CreER^T2^* cassette consists of the *Nse*-promoter containing the entire non-coding exon I, followed by the rabbit β-globin intron II (flanked by parts of β-globin exon II and III; 3′ and 5′, respectively), and the *CreER^T2^* gene with a poly-A signal. The *Nse*-*Cre*-PCR (for protocol see section above) was designed to span the β-globin intron: the forward primer binds to the *Nse*-exon region, the reverse primer to the *Cre*-coding region (see **Fig. 1B**). Amplification of genomic DNA yields a 916 bp fragment, the same PCR using cDNA as a template results in a 343 bp fragment (**Fig. 1C**).

### Breeding of *Nse*-*CreER^T2^* Transgenic Mice with Reporter Lines and Tamoxifen Treatment

Mice were crossed with the reporter mouse lines *Rosa26-LacZ* (abbreviated *LacZ: Gt(ROSA)26Sor^tm1Sor^* [13]) or *R26R-td-tomato-mEGFP* (abbreviated *mTmG*: *Gt(ROSA)26Sor^tm4(ACTB-tdTomato,-EGFP)Luo^* [14]. P3 mice were once intraperitoneally injected with 300 µg Tamoxifen (Sigma, T5648, 18 mg/ml in sunflower oil and 10% ethanol) and sacrificed three weeks later (P23) to analyze Cre recombinase activity. Adult mice (>2 months) were intraperitoneally injected with 135 µg Tamoxifen/g bodyweight on five subsequent days. Recombination was analyzed not earlier than 10 days after the injection.

### Perfusion and Sectioning

Transcardial perfusion started with PB (0.1 M phosphate buffer, pH 7.4) for 5 min (ca. 50 ml), followed by fixative solution with 4% paraformaldehyde in PB (PFA) for an additional 5 min (ca. 50 ml). Removed brains were postfixed in 4% PFA at 4°C overnight and stored in PB with 0.02% NaN_3_ at 4°C. Brains were embedded in 5% agarose (in PB) and 50 µm sections were cut with a Leica VT 1000S Vibratome.

### Immunohistochemistry and LacZ Staining

Sections were blocked for 2 h in PB-Tx (PB with 0.1% Triton X) containing 10% donkey serum. To amplify the mGFP (membrane-tagged GFP) signal, sections were incubated with anti-GFP antibody (rabbit, Clontech) at 4°C overnight. After three washing steps with PB-Tx, slices were incubated with the secondary anti-rabbit Alexa Fluor 488-coupled antibody (donkey, Invitrogen) for 2 h at room temperature. Slices were washed once with PB-Tx containing DAPI (1∶10.000, Applichem) and twice with PB-Tx, each washing step 20 min at room temperature, and finally mounted with Mowiol.

For co-immunohistochemistry of GFP-positive cells and neuronal marker proteins, slices were first blocked for 2 h in PB-Tx containing 10% goat serum and 10% Avidin (Vector Laboratories) at room temperature. The primary antibodies anti-GFP (rabbit, Clontech; for co-detection with GFAP: mouse, Millipore) and anti-NeuN (mouse, Chemicon), anti-GFAP (rabbit, Dako Cytomation), anti-Calbindin (mouse, Swant), or anti-Map2 (mouse, Leinco) were incubated in PB with 10% Biotin (Vector Laboratories) for 48 h at 4°C. After washing in PB-Tx the slices were incubated with secondary biotinylated antibody directed against the neuronal marker antibody (anti-mouse/anti-rabbit) for 2 h at room temperature. Incubation with AMCA-Avidin D (goat, Vector Laboratories; to detect biotin) and the secondary Alexa Fluor 488-coupled antibody (donkey, Invitrogen; directed against anti-mouse/anti-rabbit GFP) in PB-Tx was performed, followed by an additional washing step for 2 h at room temperature. Anti-Parvalbumin antibody (rabbit, Swant) was used for co-immunohistochemistry with anti-GFP (mouse, Clontech) and detected with Alexa Fluor 350 and 488 secondary antibodies (goat, Invitrogen). After a final washing step the slices were mounted with Mowiol.

For LacZ staining first the slices were incubated on ice, twice (5 min and 10 min) with 2 mM MgCl_2_ in PB, and once for 10 min in Detergent Rinse (2 mM MgCl_2_, 0.01% Natriumdeoxycholate, and 0.02% NP40 in PB). Afterwards, the slices were incubated in staining solution (Detergent Rinse plus 5 mM Potassium ferricyanide, 5 mM Potassium ferrocyanide, and 1 mg/ml x-Gal) for 3 h at 37°C. After washing, the slices were mounted with Mowiol. Pictures were imaged on a Zeiss Axioplan 2 microscope.

## Results

### Generation of an *Nse*-*CreER^T2^*-Line

To enable an inducible, neuron-specific Cre recombination of LoxP-flanked target-regions, an *Nse*-*CreER^T2^* cassette was designed and microinjected into oocytes to generate *Nse*-*CreER^T2^* transgenic mice. The *Nse*-promoter contains 1.8 kb DNA of the rat neuron-specific enolase (NSE) gene promoter, described in Cinato et al. [11], including 43 bp of the first noncoding exon. In the *Nse-CreER^T2^* construct the promoter is followed by the rabbit β-globin intronic enhancer sequence (spanning intron II and containing 18 bp of the 5′ flanking exon II and 53 bp of the 3′ flanking exon III, see **Fig. 1B**) to increase expression levels of the downstream-located *CreER^T2^*-gene.

To verify the function of the *Nse*-*CreER^T2^* construct *in vitro* prior to the generation of transgenic mice, neuroblastoma cells (N2A) were transiently co-transfected with pGS-*Nse*-*CreER^T2^* and a test-plasmid containing a LoxP-flanked region. Recombination was verified by PCR and only occurred in co-transfected cells that were treated with 4-Hydroxytamoxifen (data not shown).

Pronucleus injection of the linearized construct yielded one founder mouse. To detect the *CreER^T2^* transcript by RT-PCR, a primer pair was designed that spans the β-globin intron II, allowing us to distinguish the PCR product of the genomic *CreER^T2^* gene from the cDNA by their transcript sizes (916 versus 343 bp, respectively, **Fig. 1C**).

### Pattern of Inducible Cre Activity in the Generated Mouse Line

To analyze the expression pattern and function of Cre recombinase, different reporter lines are available, carrying LoxP modified marker-genes. Here, the *mTmG* [14] and the *LacZ* (*Rosa26-LacZ* [13]) reporter strains were used. In *mTmG* mice the CMV β-actin enhancer-promoter (CAG [15]) and two adjacent marker-genes are targeted into the *Rosa26* locus. The first gene, encoding a membrane-tagged Tomato fluorescence protein (mT or mTomato), is flanked by LoxP sites and will be expressed as long as recombination does not occur. After Cre-mediated excision of *mTomato* the second gene, encoding membrane-tagged GFP (mG or mGFP), will be transcribed, switching the cell from producing a red to a green signal. To amplify the mGFP signal an anti-GFP antibody was used.

**Figure 2:**
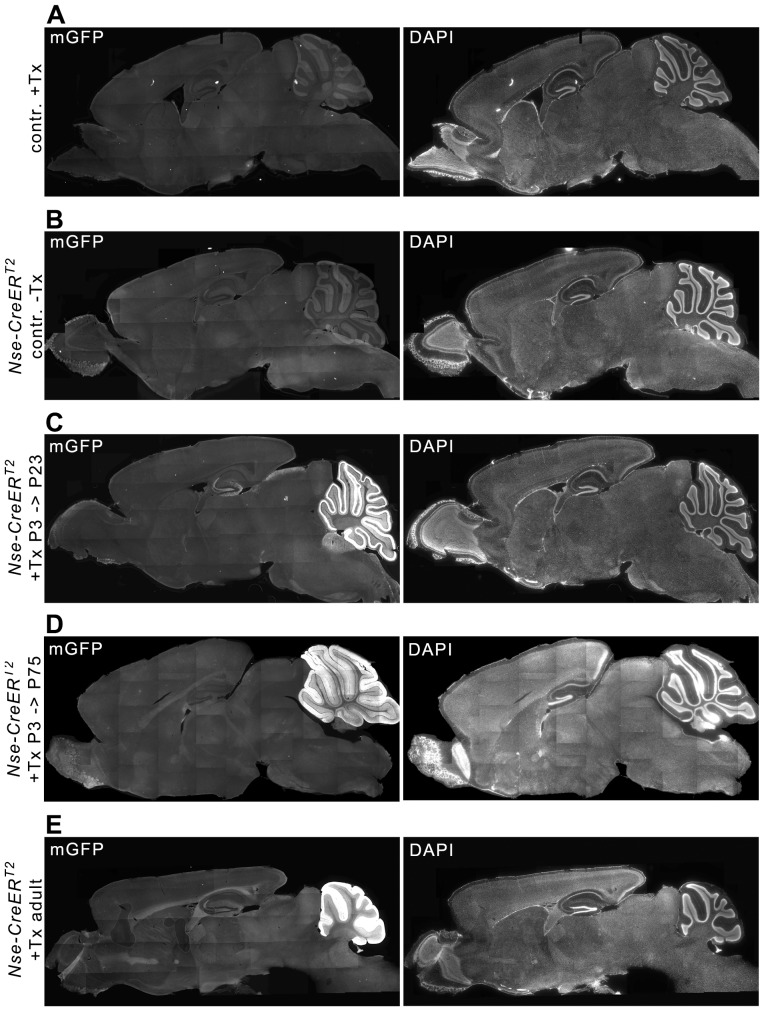
Tamoxifen-induced CreER^T2^-activity in the juvenile and mature brain of P3- and adult-injected *Nse-CreER^T2^;mTmG* mice. In control mice not carrying a *CreER^T2^*-tansgene (A) or in *CreER^T2^*-transgenic mice without tamoxifen injection (B), the mGFP reporter was not labeled. C-E: Tamoxifen injection of P3 (C, D) and adult (E, section more lateral, compared to A-D) mice led to a strikingly similar fluorescence pattern of mGFP in the juvenile (P23 in C) or mature (D and E) brain. mGFP fluorescence was found to be most intense in the cerebellum. Less intense staining of cells and fibers was observed in the hippocampus, and some fibers in nuclei of the midbrain and pons showed mGFP-immunoreactivity.

In *Nse-CreER^T2^*;*mTmG* mice injected with tamoxifen at P3 or at adult age (>2 months) broad mGFP-immunoreactivity was found in the developing (P3-injected → P8), juvenile (P3-injected → P23), and mature (P3-/adult-injected → adult) cerebellum (**Fig. 2C-E** **and** **5**). Recombination was absent in tamoxifen-injected *mTmG* reporter animals not carrying the *Cre*-transgene (**Fig. 2A**) and in non-injected *Nse-CreER^T2^*;*mTmG* mice (**Fig. 2B**).

**Figure 3:**
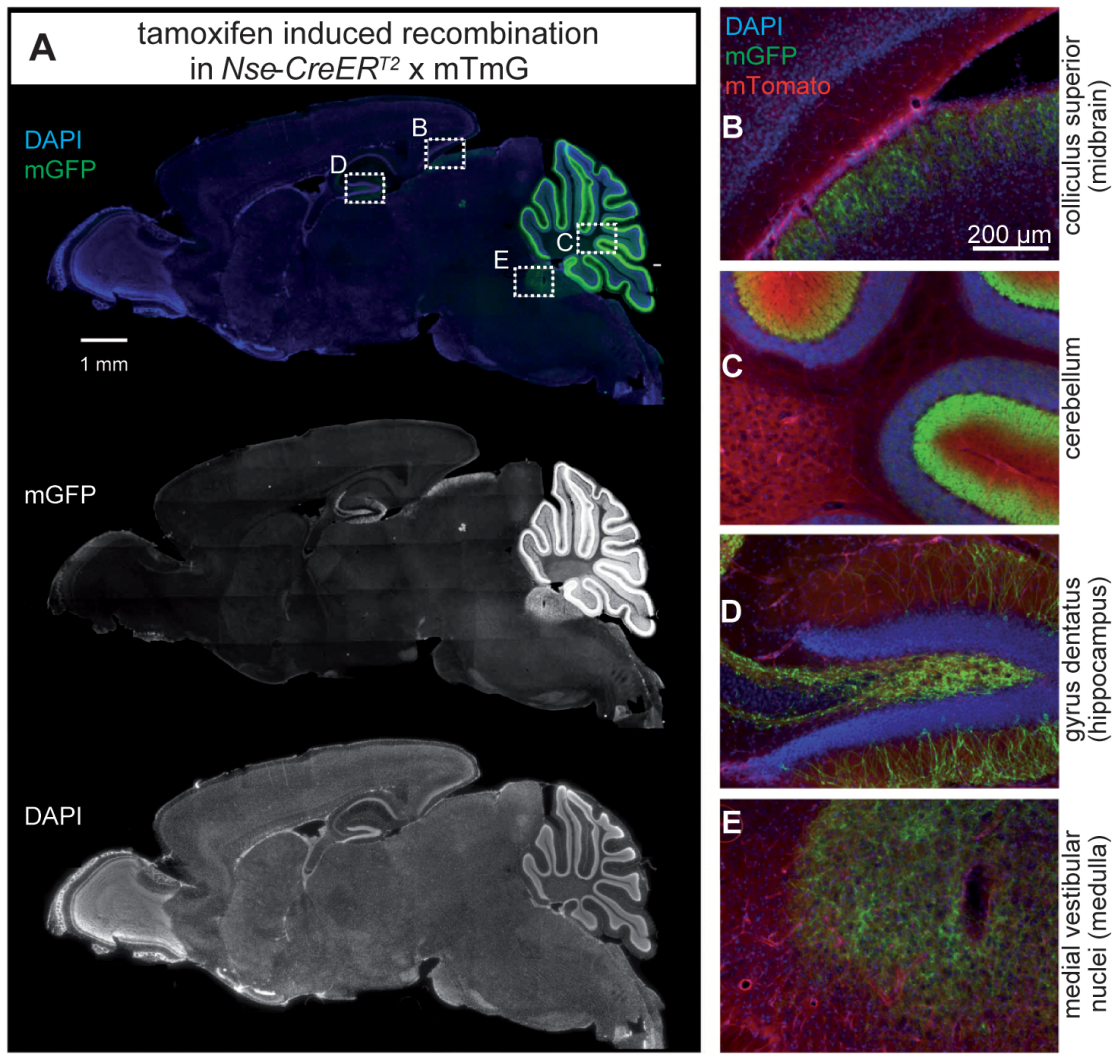
CreER^T2^-activity in *Nse-CreER^T2^;mTmG* after tamoxifen injection. Sagittal section showing mGFP-immunoreactive cells (green, A) indicating that recombination has occurred (mGFP/single channel in the middle) and DAPI-stained nuclei (blue/single channel at the bottom). Enlargements of the framed rectangles in sections B to E are shown on the right: (B) fibers in a region of the colliculus superior in the midbrain, (C) layers of the cerebellum, (D) hippocampal granule cells and their fibers, and (E) fibers in the medial vestibular nuclei (medulla) show green fluorescence. Red (mTomato) fluorescence in 1–4 shows cells where no recombination occurred.

**Figure 4:**
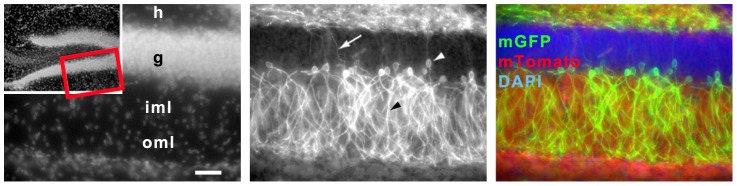
Dentate gyrus of *Nse-CreER^T2^*;*mTmG* after tamoxifen injection. Left: DAPI staining of dentate gyrus, the red-framed area is shown enlarged. Middle: mGFP-immunoreactive cells where recombination has taken place are located in the outer part of the GCL. White arrowhead points to the soma, white arrow to an axon, black arrowhead to a dendrite of GFP-immunoreactive granule cells. Right: Merged picture showing nuclei in blue (DAPI), cells without recombination in red (mTomato autofluorescence) and cells where recombination occurred in green (mGFP-immunoreactive cells). Scale bar  = 50 µm.

In addition to the cerebellum, dispersed recombination was detected in the hippocampus: individual granule cells of the dentate gyrus and their mossy fibers projecting into the hilus were labeled (**Fig. 3D** **and** **Fig. 4**). Additional mGFP-positive fibers were found in the colliculus superior (midbrain, **Fig. 3B**) and medial vestibular nuclei (medulla, **Fig. 3E**).

### Distribution of Cre Recombinase Activity in the Cerebellum

**Figure 5:**
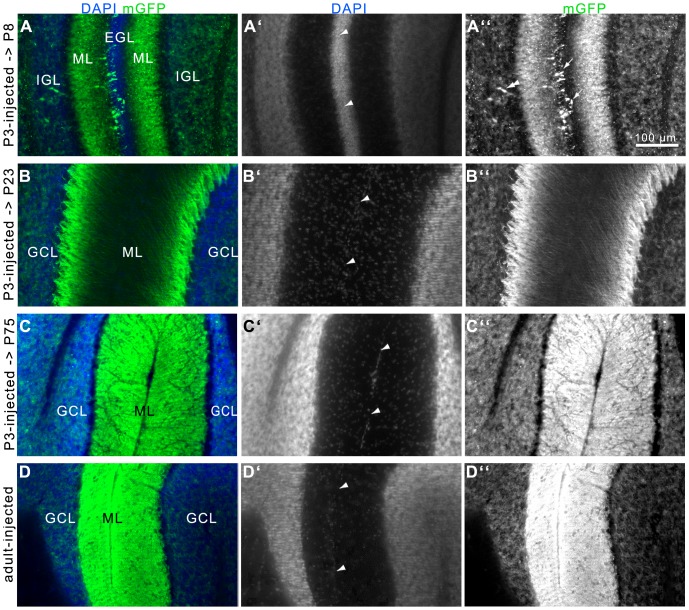
Development of the GCL and ML in the cerebellum of *Nse-CreER^T2^;mTmG* mice after tamoxifen-induction. A-C: P3-injected mice were killed at P8 (A), P23 (B), or P75 (C). Pictures show the EGLs (A) or MLs (B-D) next to a fissure (arrowheads in A′-D′) of two lobules. A: At P8 some mGFP-postive GCPs are found in the EGL (arrows in A″) and a substantial amount of GCs are labeled in the IGL. Double arrowhead points to a presumably migrating cell with leading/trailing processes. Structures in the ML are immunoreactive as well. B: At P23 cells are labeled in the GCL, and structures are labeled in the ML. C: At P75, the labeling of GCs in the GCL is comparable to P8 and P23, but the structures in the ML show a more brightly immunolabeling, filling the entire ML. D: The mGFP labeling in the GCL and ML of an adult-injected mouse is strikingly similar to that of the P3-injected adult mouse in C. A-D  =  merge, A′-D′  =  DAPI, A″-D″  =  mGFP. Scale bar  = 100 µm.

In adult mice the GFP signal was detected in the entire region of the cerebellar ML and GCL when injected with tamoxifen at P3 or at adult age. In the GCL, the individual GC-somata were visible, whereas the staining in the ML was too intense to differentiate between individual structures (**Fig. 5C and D**). However, in the P3-injected early postnatal (P8) and juvenile (P23) mice a less intense fluorescence allowed the identification of fibrous structures (**Fig. 5A and B**) extending from the GCL into the ML. In P8 animals the trailing and/or leading processes of GCs are labeled in the inner EGL, ML, and IGL (**Fig. 5A** **and** **6**), demonstrating that some postmitotic GCPs express *CreER^T2^*. At P23, fluorescent somata were only seen in the GCL, and in the ML fibrous structures were labeled. In contrast, mGFP fluorescence of GC somata in the IGL/GCL was of comparable intensity and density in all mice (**Fig. 5 A-D** **; Fig. S1**).

**Figure 6:**
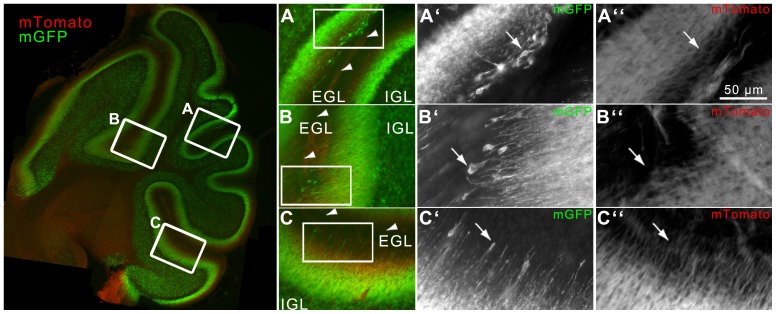
mGFP-labeled, postmitotic migrating GCPs in the EGL with trailing and leading processes at P8. Left: P3-injected P8 cerebellum, mGFP  =  cells after recombination, mTomato  =  cells without recombination. Frames A-C are shown enlarged, A′-C′  =  further enlargement to show mGFP-immunoreactive GCPs in the inner EGL, A″-C″  =  mTomato. Arrowheads in A-C point to the fissure between two lobules. Arrows presumable point to tangentially (A′, with leading and trailing processes) or radially (B′, C′, with leading and/or trailing processes) migrating GCPs in the EGL.

### Cre-Mediated Recombination is Restricted to Cerebellar GCs

**Figure 7:**
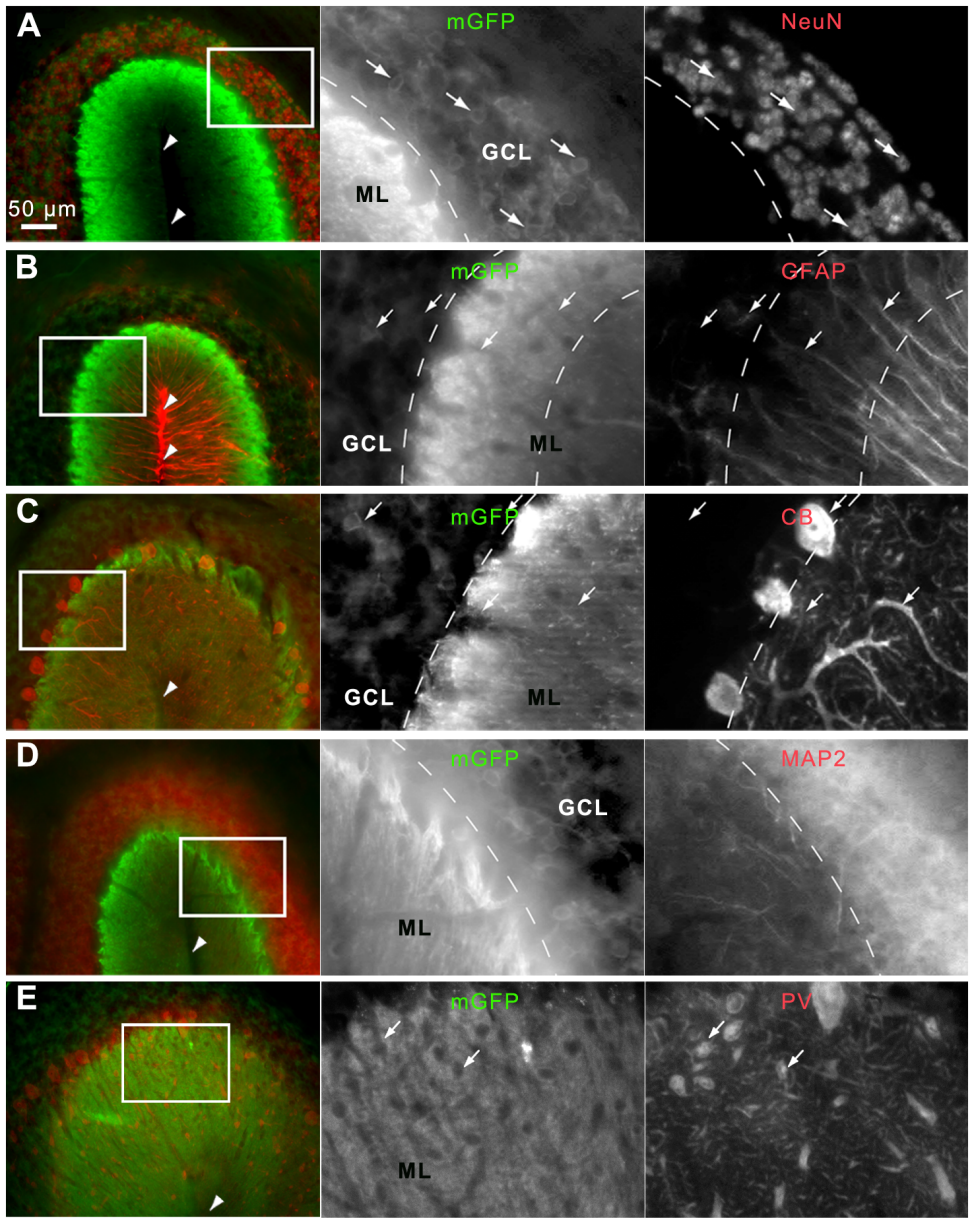
Cerebellar lobules of tamoxifen-injected *Nse-CreER^T2^;mTmG* mice labeled for different neuronal marker proteins. A-E: mGFP-immunoreactivity is shown in green (mGFP), immunoreactivity for different cell type specific markers is shown in the red channel (labeled with blue fluorescence). Arrowheads point to the fissure between two lobules. A: NeuN (in the cerebellum a GC-specific marker), B: GFAP (produced by cells of glial lineage), C: Calbindin (CB, produced by Purkinje cells), D: MAP2 (located in neuronal dendrites), and E: Parvalbumin (PV, expressed by Purkinje cells and ML interneurons). NeuN was mainly found in the GCL and cells were co-immunoreactive for GFP. GFAP-labeled structures showed no GFP-signal, nor did Purkinje cells or the interneurons of the ML. MAP2-positive structures were densely immunostained in the GCL, representing the dendrites of GCs. Scale bar  = 50 µm.

To clarify the identity of the mGFP-labeled fibrous structures, a Calbindin and GFAP co-staining, labeling Purkinje cell dendrites and the Bergmann glial fibers, was performed. Neither Calbindin- nor GFAP-positive structures showed a co-immunoreactivity with mGFP (**Fig. 7B,C**). NeuN-immunoreactivity was found in the nuclei of mGFP-postive cells (**Fig. 7A** **and Fig. S1**). NeuN is an established GC-marker; it is not expressed in Purkinje cells [16], interneurons of the ML, and several other non-GC cerebellar neurons [17]. When injected at P3 and examined at P8 a small fraction of NeuN-positive cells in the EGL expressed mGFP but several GCs in the IGL were co-labeled (**Fig. S1A**). In the more juvenile and mature brain of P3-injected animals, the majority of NeuN-stained GCs showed mGFP expression (**Fig. S1B and C**). In the P8 and P23 brain some mGFP-positive structures in the IGL or GCL, respectively, are not surrounding NeuN-positive nuclei (**Fig. 7A** **and Fig. S1A and B**) fewer of these structures were found in the adult brain (**Fig. S1C**). MAP2-immunoreactivity, visualizing dendritic structures, overlapped with mGFP in the GCL (**Fig. 7D**). Lack of co-immunostaining of mGFP with Parvalbumin, which labels stellate and basket cells in the ML [18], [19], excludes the possibility that these interneurons express *CreER^T2^* (**Fig. 7E**).

**Figure 8:**
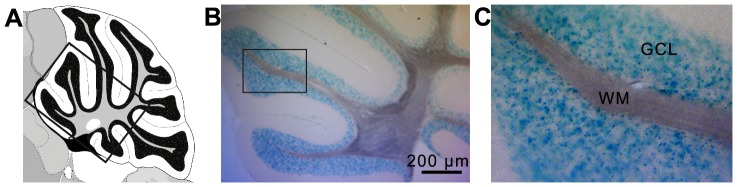
Cerebellar LacZ-staining of *Nse-CreER^T2^;LacZ* mice. A: Schematic overview, B: LacZ staining (blue) is restricted to the GCL, one lobule is shown enlarged in (C). WM  =  white matter.

To further clarify that recombination is restricted to GCs in the cerebellum, another reporter line was used. In the *LacZ* reporter line a *LacZ* gene, with a preceding floxed stop cassette, is targeted into the *Rosa26* locus, ensuring that β-Gal is expressed only after recombination. The gene product hydrolyzes X-gal, leading to the precipitation of 4-chloro-3-brom-indigo in *LacZ* expressing cells. LacZ staining of an adult-injected *Nse*-*CreER^T2^* mouse carrying the *LacZ* reporter demonstrated that the blue precipitate was restricted to the somata of cells in the cerebellar GCL (**Fig. 8**), whereas in the ML staining was absent. To further ensure that Cre-activity is restricted to the GCL, a co-immunostaining against Parvalbumin and β-Gal was performed: β-Gal labeling was absent in the ML and did not co-stain with Parvalbumin labeled interneurons or with Purkinje cells (**Fig S2)**.

## Discussion

The *Nse-CreER^T2^* transgenic mouse line described here was established with the purpose to provide a neuron-specific, temporally inducible recombination of LoxP-flanked genomic regions. To achieve this, a promoter of the neuron-specific enolase (*Nse*) gene containing a 1.8 kb DNA-fragment upstream of the rat *Nse*-coding region was used. *Nse*-transgenic mouse lines using this promoter have been described where a *Cre* or *LacZ* transgene was ubiquitously expressed in neurons [11], [20]. However, the expression levels and pattern of *Nse*-promoter-driven *Cre* and *LacZ* transgenes significantly varied between the founder lines established by Cinato et al.[11], Forss-Petter et al.[20], and Kwon et al.[21]; and several founder lines showed a dispersed neuronal expression as result of position effects that are common in transgenic animals [22], [23]. In our *NSE-CreER^T2^* line, CreER^T2^ was almost exclusively produced in the GCs of the cerebellum.

### Pattern of Cre Recombinase Activity After Tamoxifen Injection Outside the Cerebellum

Our *Nse*-*CreER^T2^* line shows a distinct expression of *CreER^T2^* in the cerebellum that is strikingly restricted to cells in the GCL. The hippocampus showed restricted CreER^T2^ mediated recombination, too. Cre recombinase activity was observed in the outer granule cell layer of the dentate gyrus. Dentate gyrus granule cells in the outermost layer are the most mature neurons, while granule cell progenitors reside in the subgranular zone at the inner side of the granule cell layer [24]. In *Nse-CreER^T2^;mTmG* mice we could not see any obvious differences in the amount and location of mGFP-postive granule cells in the gyrus dentatus of P3 and adult-injected animals.

In addition, immunoreactive fibers in different brain regions were found, e.g. in the superficial-most layers of the superior colliculus, where retinocollicular projections terminate [25], [26]. Some mGFP-postive fibers were also found in the medial vestibular nuclei of the medulla that are traversed by neurons of the vestibular nerve terminating in the cerebellar nuclei. In general, the same pattern of Cre activity was found in adult-injected animals and young (P8), juvenile (P23), or adult (P75) P3-injected animals.

### Initiation of *CreER^T2^* Expression in Cerebellar GCPs and Mature GCs

The proliferation of GCP in mice starts shortly before birth and lasts until P15 [5]. Migration and differentiation continues until adulthood [6]. Depending on the age of the animal at the time of sacrifice a difference in the appearance of mGFP-postive cerebellar fibers was noted, whereas the density of mGFP-postive GC-somata in the GCL was comparable. In P3- or adult-injected mice, analyzed at two months or older, the immunoreactivity of fibers filled the entire ML, whereas in P3-injected mice sacrificed at P8 and P23 the fibrous structures extending from the inner to the outer region of the ML thinned out. Whereas in the P23 cerebellum all GCs are located in the mature GCL, at P8 GCPs are still present in the EGL, before migrating into the IGL. In P3-injected P8 animals GCPs are labeled in the EGL, demonstrating that transcription initiation of the *CreER^T2^* transgene starts in GCPs. As only a few GCPs in the inner EGL are labeled, *CreER^T2^* expression is not yet active in proliferating GCPs. mGFP-labeled cells in the inner EGL extend radial leading processes which are used by GCPs to migrate along radial Bergmann glial fibers [27], [28]. GCPs that seem to pursue a tangential route of migration were seen, too (**Fig. 6A**). The low amount of mGFP-labeled GCPs in the EGL at P8 can be the result of the restricted effective period of tamoxifen action (injected at P3): due to different metabolic activity, tamoxifen-induced recombination events in the embryo are restricted to a short time frame (up to 48 h), compared to several days/up to weeks in the adult brain [29]. With these data one can estimate that P3-injected tamoxifen loses its effect hours/days before P8. Thus, it can be assumed that at P8 more *CreER^T2^* expressing postmitotic GCPs are present than are effectively labeled by the CreERT2 reporter gene product.

Depending on the age of the mice the appearance of mGFP-labeled GC processes ascending into the ML varies. GCs ascend their axons into the ML, where they bifurcate orthogonally into parallel fibers. Parallel fibers are oriented transversally and are therefore cut orthogonally in sagittal sections. They innervate the dendritic trees of Purkinje cells that in turn are orientated in the sagittal plane. The developmental organization of the ML is well described, especially with regard to the maturation of the Purkinje cell dendrites. The dendritic tree of Purkinje cells expands massively between P7 and P30, reaching its lateral boundaries at P12–15 and expanding to the borders of the pia mater until P30 [30]. Recent findings show that this organization is strictly confined to sagittal planes until the fourth postnatal week [31]. Furthermore, dendritic arborization is dependent on GCs and their parallel fibers [32]–[34]. Finally the dendritic tree at P23 does not yet fully expand to the pia mater, as do the innervating parallel fibers. In the P8 and P23 cerebellum, we observe fiber structures in the ML that can be related to leading processes and/or ascending axons from migrating and/or matured GCs. We could not clearly define mGFP-labeled parallel fibers in the ML, which can either be due to the orientation of the sections (sagittal) or due to the distribution of mGFP in the axons. At P8 mGFP is highly present in the leading and trailing processes of migrating GCs. However, in the adult cerebellum the cell bodies of mature GCs are restricted to the GCL and only their ascending axons and parallel fibers extend into the ML, which is brightly labeled with mGFP at this point. Hence the ascending axons of GCs and/or their parallel fibers are mGFP-labeled in the mature cerebellum. For a better visualization of cell morphology, reporter strains expressing non-membrane tagged fluorescent proteins like the Ai6- (ZsGreen) or Ai14- (tdTomato) reporter [35] can be considered.

### Coverage of GCs Expressing *CreER^T2^*

With both reporters we could detect that abundant recombination occurred in the GCL in all lobules. NeuN immunoreactivity labels the nucleus of neurons. We used NeuN as an established GC-specific marker [17]: Co-immunohistochemistry of mGFP and NeuN – even though tamoxifen was injected at P3 – showed an extensive overlap in the IGL/GCL of animals sacrificed at different ages (**Fig. S1**). Some mGFP-positive structures in the IGL and GCL are not surrounding NeuN-positive nuclei. Fewer of these structures are found in the adult (P75) compared to the pup (P8) and juvenile (P23) cerebellum. Proliferation of GCs lasts until P15, then they migrate to their final position in the IGL where the dendritic development occurs to establish synaptic connections to mossy fiber axons. During this transformation, there is a phase of production of provisional dendrites, followed by retraction and pruning of processes that last until adulthood where the distal ends of the remaining dendrites finally have a claw-like morphology [36], [37]. These claw-like structures are part of synaptic glomeruli that are located in the GCL and are composed of large mossy fiber axon terminals, surrounded by dozens of GC dendrites, as well as Golgi cell axons and dendrites [37]–[39]. mGluR2 Immunostaining of Golgi cells illustrates their cell bodies and their processes terminating in glomeruli [40]. Whereas the Golgi cells are larger in size (around 20 µm), the diameter of a glomerulus (5–10 µm) is similar to the GC diameter (6–8 µm). The mGFP-positive structures not surrounding NeuN-labeled nuclei can be attributed to synaptic glomeruli and (in the developing cerebellum) provisional dendrites. In contrast, the size and shape of mGFP-labeled structures clearly resembles somata of GCs (**Fig. S1**).

While the GC morphology with the membrane-tagged fluorescent reporter protein mGFP was difficult to track, the LacZ reporter clearly showed that CreER^T2^-positive cells were restricted to the GCL. However, at this point we cannot clearly say if all GCs express *CreER^T2^* since GCs are very tiny and densely packed, which makes it difficult to differentiate between single cells to quantify the coverage.

Of note, other transgenic mouse lines with an inducible Cre recombinase that is active in cerebellar GCs have been described before; however, none of them cause a specific and inducible recombination in the GCs of all lobules in both the developing and adult cerebellum [41]–[43].

## Conclusion

The described *Nse-CreER^T2^* transgenic mouse is a powerful tool to address different aspects of cerebellar development. The line has been deposited at the Jackson laboratories (Jax Stock 022763). Mature GCs and their fibers as well as GCPs in the inner EGL can be fate-tracked at different developmental time points by using different reporter lines. Additionally individual floxed genes can be knocked out to study their function in a time-dependent and GC-specific manner. This will allow to tackle questions concerning maturation of the cerebellum and the assembly of the network of GC ascending axons and parallel fibers in the ML. In addition, the role of distinct presynaptic proteins in synaptic plasticity can be addressed using this mouse model, since long term depression is a prominent type of synaptic plasticity at the parallel fiber-Purkinje cell dendrite synapses and a widely accepted model for storing motor memories [44].

## Supporting Information

Figure S1 Cerebellar lobules of P3-injected *Nse-CreER^T2^;mTmG* mice co-labeled for NeuN and mGFP.
(TIF)

Figure S2 β-Gal immunoreacitivity was absent in the ML and did not co-label with Parvalbumin.
(TIF)

Text S1 Supplementary Materials and Methods, Figure Legends S1 and S2.
(DOCX)
