# Supplementary figures and images for: Generation and Characterization of an Nse-CreERT2 Transgenic Line Suitable for Inducible Gene Manipulation in Cerebellar Granule Cells
Source: PLoS One. 2014 Jun 20;9(6):e100384. doi: 10.1371/journal.pone.0100384 (PMC4065071; doi:10.1371/journal.pone.0100384)

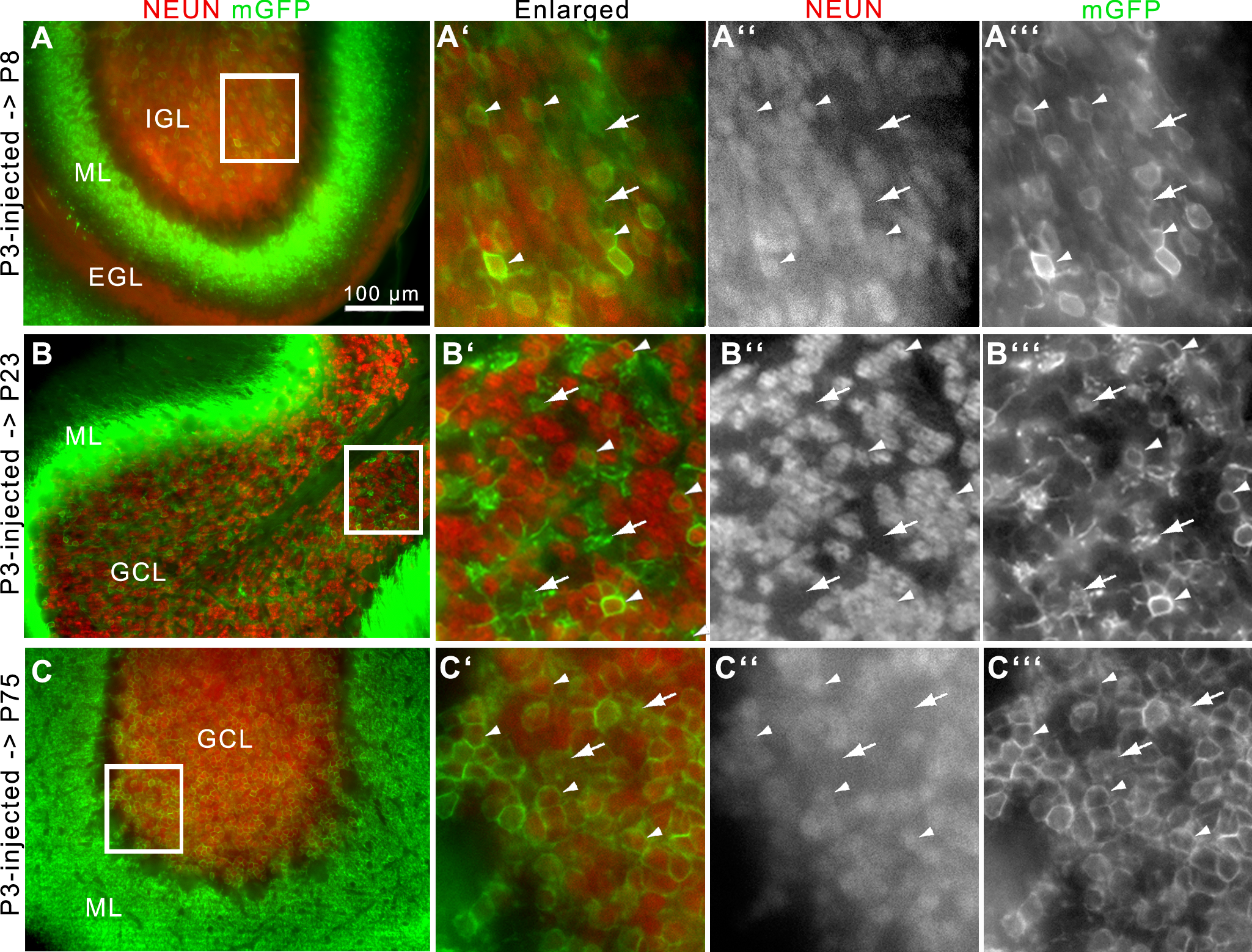

Supplement: Figure S1 — Cerebellar lobules of P3-injected Nse-CreERT2;mTmG mice co-labeled for NeuN and mGFP. (TIF) [file pone.0100384.s001.tif]

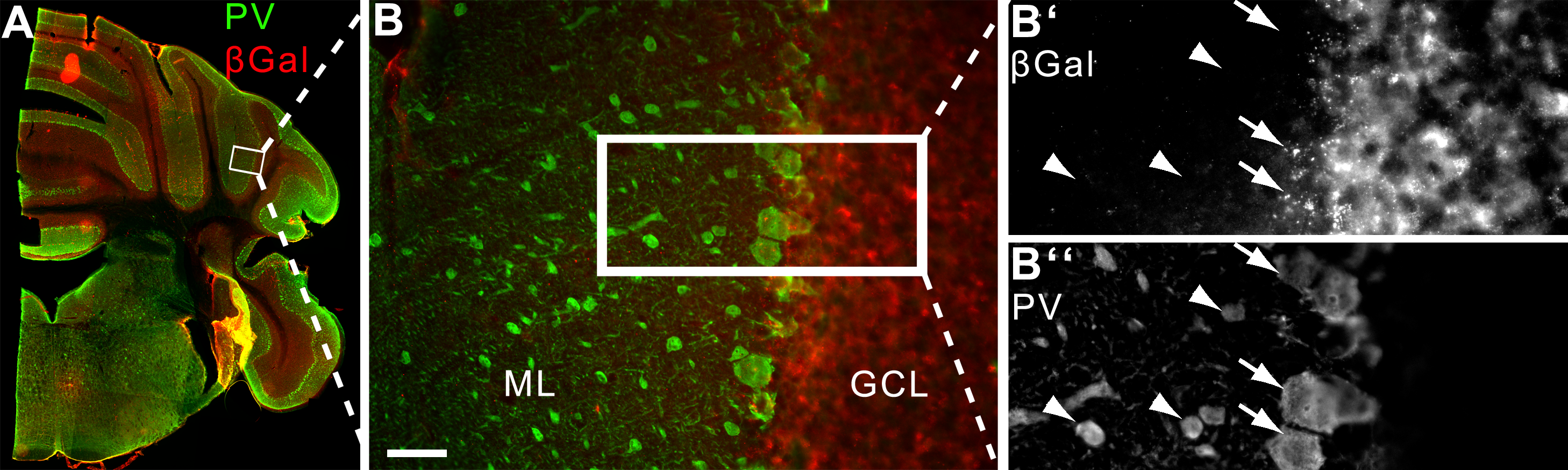

Supplement: Figure S2 — β-Gal immunoreacitivity was absent in the ML and did not co-label with Parvalbumin. (TIF) [file pone.0100384.s002.tif]
