# Supplementary material for: Generation and Characterization of an Nse-CreERT2 Transgenic Line Suitable for Inducible Gene Manipulation in Cerebellar Granule Cells
Source: PLoS One. 2014 Jun 20;9(6):e100384. doi: 10.1371/journal.pone.0100384 (PMC4065071; doi:10.1371/journal.pone.0100384)
Supplement: Text S1 — Supplementary Materials and Methods, Figure Legends S1 and S2. (DOCX) [file pone.0100384.s003.docx]

**Supplementary Materials and Methods:**

Co-immunohistochemistry was performed as described in the main manuscript. Anti-β-Gal antibody (mouse, Cell Signaling, 1:1000) was used with anti-Parvalbumin (rabbit, Swant, 1:1000).

**Supplementary Figure S1: Cerebellar lobules of P3-injected *Nse-CreER^T2^;mTmG* mice co-labeled for NeuN and mGFP.** A-C: mGFP-immunoreactivity is shown in green (mGFP), immunoreactivity for the GC-marker NeuN is illustrated in red (detected with a blue fluorescent secondary antibody; Alexa Fluor 350). All three mice in A-C were injected with tamoxifen at P3 and examined at P8 (A); at P23 (B), or at P75 (C). Framed regions are shown enlarged in A’-C’; single channels in A’’-C’’ (NeuN) and A’’’-C’’’ (mGFP). Arrowheads point to single co-labeled GCs, arrows point to GFP immunoreactive structures not surrounding NeuN-stained nuclei.

**Supplementary Figure S2: β-Gal immunoreacitivity was absent in the ML and did not co-label with Parvalbumin.** A: Coronal section of an NSE-CreER^T2^;R26-LacZ mouse brain hemisphere. Tamoxifen was injected at adult age. β-Gal (red) labels cells where Cre-mediated recombination has occurred, Parvalbumin (PV, green) labels interneurons of the ML and Purkinje Cells. B: Magnification showing the ML with Parvalbumin labeled interneurons (arrowheads in B’ and B’’), the GCL with β-Gal immunoreactive GCs, and in between the larger Parvalbumin-immunoreactive Purkinje cells (arrows). Scale bar 50 µm.
